# Supplementary material for: Genetic Diversity and Synergistic Modulation of Salinity Tolerance Genes in Aegilops tauschii Coss
Source: Plants (Basel). 2021 Jul 7;10(7):1393. doi: 10.3390/plants10071393 (PMC8309352; doi:10.3390/plants10071393)
Supplement: Supplementary file 1 [file plants-10-01393-s001.zip › plants-1239616-supplementary.pdf]

Table S1. Populations informations.

| Code | Population | Collection location | Province |
|---|---|---|---|
| 1 | HL-1 | Qianyuan Village, Kongyu Guo Town, Lucheng County, Luohe City, Henan Province China | Henan |
| 2 | SP-1 | Guandao Village, Houzhen Township, Lantian County, Weinan City, Shaanxi Province China | Shaanxi |
| 3 | SP-2 | Guandao Village, Houzhen Township, Lantian County, Weinan City, Shaanxi Province China | Shaanxi |
| 4 | SP-3 | Guandao Village, Houzhen Township, Lantian County, Weinan City, Shaanxi Province China | Shaanxi |
| 5 | SNP-1 | Dongwu Village, Wenkou Town, Daiyue County, Tai'an City, Shandong Province China | Shandong |
| 6 | SNP-2 | Dongwu Village, Wenkou Town, Daiyue County, Tai'an City, Shandong Province China | Shandong |
| 7 | SNP-3 | Dongwu Village, Wenkou Town, Daiyue County, Tai'an City, Shandong Province China | Shandong |
| 8 | SNP-4 | Dongwu Village, Wenkou Town, Daiyue County, Tai'an City, Shandong Province China | Shandong |
| 9 | SNP-5 | Dongwu Village, Wenkou Town, Daiyue County, Tai'an City, Shandong Province China | Shandong |
| 10 | SNP-6 | Dongwu Village, Wenkou Town, Daiyue County, Tai'an City, Shandong Province China | Shandong |
| 11 | SAXP-1 | Longmen Village, Lumenkou Park Town, Hejin County, Yuncheng City, Shanxi Province China | Shanxi |
| 12 | SAXP-2 | Xiwang Village, Chaijia Town, Hejin County, Yuncheng City, Shanxi Province China | Shanxi |
| 13 | SAXP-3 | Xiwang Village, Chaijia Town, Hejin County, Yuncheng City, Shanxi Province China | Shanxi |
| 14 | SAXP-4 | Xiwang Village, Chaijia Town, Hejin County, Yuncheng City, Shanxi Province China | Shanxi |
| 15 | HP-1 | Qugou Village, Guanglu Town, Ci County, Bengbu City, Hebei Province China | Hebei |
| 16 | HP-2 | Qugou Village, Guanglu Town, Ci County, Bengbu City, Hebei Province China | Hebei |
| 17 | HP-3 | Qugou Village, Guanglu Town, Ci County, Bengbu City, Hebei Province China | Hebei |
| 18 | HP-4 | Qugou Village, Guanglu Town, Ci County, Bengbu City, Hebei Province China | Hebei |
| 19 | HP-5 | Qugou Village, Guanglu Town, Ci County, Bengbu City, Hebei Province China | Hebei |
| 20 | HP-6 | Qugou Village, Guanglu Town, Ci County, Bengbu City, Hebei Province China | Hebei |
| 21 | HP-7 | Qugou Village, Guanglu Town, Ci County, Bengbu City, Hebei Province China | Hebei |
| 22 | HP-8 | Qugou Village, Guanglu Town, Ci County, Bengbu City, Hebei Province China | Hebei |
| 23 | HP-9 | Qugou Village, Guanglu Town, Ci County, Bengbu City, Hebei Province China | Hebei |
| 24 | HP-10 | Qugou Village, Guanglu Town, Ci County, Bengbu City, Hebei Province China | Hebei |
| 25 | HP-11 | Qugou Village, Guanglu Town, Ci County, Bengbu City, Hebei Province China | Hebei |
| 26 | HP-11 | Qugou Village, Guanglu Town, Ci County, Bengbu City, Hebei Province China | Hebei |
| 27 | SNP-7 | Huangzhaizi Village, Dingyuanzhai Township, Guan County, Liaocheng City, Shandong Province China | Shandong |
| 28 | SNP-8 | Huangzhaizi Village, Dingyuanzhai Township, Guan County, Liaocheng City, Shandong Province China | Shandong |
| 29 | SNP-9 | Huangzhaizi Village, Dingyuanzhai Township, Guan County, Liaocheng City, Shandong Province China | Shandong |
| 30 | SAXP-5 | Shaqiao Village, Liucun Town, Yaodu District, Linyi City, Shanxi Province | Shanxi |
| 31 | HL-2 | Sanxiaoying Village, Huqiao Town, Hui County, Henan Province China | Henan |
| 32 | HL-3 | Fanyu Village-1, Gaosi Town, Xiangcheng City, Henan Province China | Henan |
| 33 | HL-4 | Fanyu Village, Gaosi Town, Xiangcheng City, Henan Province-2 China | Henan |
| 34 | HL-5 | Xiaoluozhuang, Dawu Township, Shangshui County, Zhoukou City, Henan Province China | Henan |
| 35 | HL-6 | Qiliying, Xinxiang City, Henan Province | Henan |
| 36 | HL-7 | Guzong Township, Yuyang District, Shangqiu City, Henan Province | Henan |
| 37 | HL-8 | Qiuhuazhuang, Jiaozuo City, Henan Province | Henan |
| 38 | HL-9 | Dazhangzhuang, Zhaocun Township, Ningling County, Shangqiu City, Henan Province | Henan |

| | | | |
|---|---|---|---|
| 39 | HL-10 | Wangjing Village, Neihuang County, Anyang City, Henan Province | Henan |
| 40 | SNP-10 | Shandong Province | Shandong |
